# Halotolerant Microorganism-Based Soil Conditioner Application Improved the Soil Properties, Yield, Quality and Starch Characteristics of Hybrid Rice under Higher Saline Conditions

**DOI:** 10.3390/plants13162325

**Published:** 2024-08-21

**Authors:** Wenyu Jin, Lin Li, Guohui Ma, Zhongwei Wei

**Affiliations:** 1 State Key Laboratory of Hybrid Rice, Hunan Hybrid Rice Research Center, Changsha 410125, China; jwy825@126.com (W.J.); maguohui@hhrrc.ac.cn (G.M.); 2 National Center of Technology Innovation for Saline-Alkali Tolerant Rice, Sanya 572024, China

**Keywords:** salt stress, grain quality, rice yield, soil nutrients

## Abstract

Soil salinity represents a significant factor affecting agricultural productivity and crop quality. The present study was conducted to investigate the effects of soil conditioner (SC) comprising halotolerant microorganisms on the soil fertility, yield, rice quality, and the physicochemical and structural properties of starch in hybrid rice under saline conditions. The experimental treatments were composed of two high-quality hybrid rice varieties, i.e., ‘Y Liangyou 957’ (YLY957) and Jing Liangyou 534 (JLY534), and two soil amendment treatments, i.e., the application of SC at control levels and 2250 kg hm^−2^, or ‘CK and SC’, respectively. The crop was subjected to a mixture of fresh and sea water (EC 11 dS/m). The results demonstrated that the application of SC significantly enhanced the rice yield under salt stress conditions owing to an increase in the number of grains per panicle. Furthermore, SC was found to be effective in improving the organic matter and soil nutrient content. Furthermore, the application of SC resulted in an improvement in antioxidant defense, higher leaf SPAD values, and greater crop biomass, as well as the translocation of photo-assimilates at the heading stage. The application of SC not only improved the milling and appearance quality but also enhanced the taste value of rice by increasing the amylose and reducing the protein content. Furthermore, the application of SC also decreased the indentations on the surfaces of starch granules and cracks on the edges of the granules. The rice varieties subjected to SC exhibited excellent pasting properties, characterized by reduced proportions of amylopectin short chains and a lower gelatinization temperature and enthalpy of gelatinization. Overall, these findings serve to reinforce the efficacy of soil conditioner as a valuable tool to improve rice productivity and sustainability with improved rice grain quality under saline conditions.

## 1. Introduction

Soil salinization is becoming increasingly severe on a global scale and has become one of the key factors limiting crop productivity [1]. According to the Food and Agriculture Organization [2], more than one billion hectares of land are affected by salinity, and the area of saline-stressed land is increasing annually owing to global warming, industrial pollution, water scarcity, and poor agricultural practices. Soil salinization is particularly prominent in arid and semi-arid regions, posing a serious challenge to local food security and agricultural production systems [3]. Rice is one of the most important food crops in the world, especially in Asia, as it is the main source of food for billions of people [4,5]. However, soil salinization significantly limits the growth, yield, and quality of rice, thus posing a serious threat to regional and global food security.

The physiological and biochemical barriers caused by salt stress severely affect the normal growth of rice [6]. For example, excessive salt in the soil can lead to leaf senescence, poor root development, reduced leaf chlorophyll content (SPAD) and photosynthesis, and insufficient nutrient absorption, ultimately resulting in decreased yields and reduced rice quality [7]. In addition, high salt concentrations can induce osmotic stress and ionic toxicity, further exacerbating the inhibition of rice growth [8]. Moreover, salt stress results in a reduced plant height and panicle length and reductions in other morphological traits of rice [9].

Salt stress can be managed to some extent by adopting cultural measures and/or crop management practices. For example, Wei et al. [10] found that the exogenous spraying of melatonin improved the K^+^/Na^+^ homeostasis and increased the salt tolerance of rice by inhibiting the absorption and translocation of Na^+^. Chen et al. [7] noticed an improvement in the root physiological and morphological traits, yield, and overall salt tolerance of rice with appropriate nitrogen application. Most of the studies focus on cultivating salt-tolerant varieties through genetic engineering and traditional breeding techniques [6]; however, there is insufficient research on the improvement and regulation of salt-affected soils through soil amendment.

In this context, the application of soil conditioners (SCs) provides a possible solution for soil salinization. There are different types of SC, such as organic soil conditioners comprising farmyard manure, green manure, humic substances, peat, and mulch and inorganic ones including gypsum, biochar, crude sulfur, organosilicone, and water-soluble polymeric (polyethylene glycol, polyvinyl alcohol) and hydrogel polymeric compounds [11,12,13]. Moreover, there are SCs composed of natural ores (such as volcanic rocks—montmorillonite, gypsum, dolomite, potassium feldspar, zeolite, etc.) and synthetic polymers (such as polymaleic acid, etc.) [12]. The SC can improve the soil’s physical properties, increase the soil aeration and water retention, and promote the activity of beneficial microorganisms, thus improving the rhizosphere environment [14,15]. Sun et al. [16] found that the application volume of conditioner products with solid waste and high levels of polymers as raw materials is relatively large, thus posing potential environmental risks. In addition, Long et al. [17] found that the cations released by the decomposition of natural mineral conditioners after application may also cause toxic effects on soil. Zheng et al. [18] showed that the continuous application of manure, maize straw, and conditioners has a positive impact on the diversity and abundance of soil microbial communities, thus enhancing the accumulation of soil carbon.

Microbial remediation is an environmentally friendly, economical, and effective method for salinization improvement [19]. Soil microorganisms are key biological factors affecting plant growth, especially the growth of halophytes in salinized soil [20]. Hu et al. [21] reported that *Bacillus subtilis* and *Bacillus Licheniformis* were the dominant microbial populations in salt-affected soil and had excellent salt tolerance abilities. Dias et al. [22] reported that *Streptomyces* promoted plant growth and metabolism under saline conditions. Moreover, Liu et al. [23] demonstrated that straw addition could increase the crop yield by improving the soil fertility, the soil aggregate stability, and the diversity of fungi. However, reports of the effects of halotolerant microorganism-based soil conditioner application in terms of crop improvement and the regulation of soil salinization are relatively scarce. Therefore, the present study aimed to explore the impact of halotolerant microorganism-based SC on the rice yield, rice quality, and soil fertility under saline soil conditions. Overall, we intend to provide effective soil management practices to alleviate the impact of soil salinization on rice production and its management.

## 2. Materials and Methods

### 2.1. Experimental Materials

A soil conditioner (SC) based on halotolerant microorganisms was specifically developed by our research team for saline–alkali land to improve the soil quality. The main effective components of the SC include a salt-tolerant synthetic microbial community (colony-forming unit count of ≥20 million colony forming units (CFUs)/g), with the carrier materials primarily consisting of 20% organic materials, such as crop straw and biochar, as well as inorganic materials including phosphogypsum, nitrogen (2.0%), phosphorus (6.5%), potassium (3.5%), silicon (10%), magnesium (10%), calcium (30%), and sugar molasses. The salt-tolerant synthetic microbial community is composed of *Bacillus subtilis*, with storage number CGMCC NO. 0395.2 and named the WH2 strain; *Bacillus Licheniformis*, with preservation number CGMCC NO. 0395.4, named the WH4 strain; *Streptomyces fradiae*, with preservation number CGMCC NO. 2166, named the WH8 strain; and *Streptomyces microflavus*, with the preservation number CGMCC NO. 7066, composed of various bacterial species, such as WH18. The manufacturing process involved mixing the microbial strains, mainly including the WH2, WH4, WH8, and WH18 strains, according to the appropriate ratio to form the WH bacterial community, which was subsequently subjected to secondary bacterial culture. Finally, the life carriers of the microbial WH bacterial communities, such as phosphogypsum, sulfur-based sugar filter mud or crop straw, phosphorus, nitrogen, potassium, silicon powder, magnesium powder, and the active bran bacteria, were mixed according to the formula, fermented at room temperature for 10–15 days, and crushed and packaged to produce a microbial fertilizer. All microbes mentioned above were provided by the Chinese Academy of Sciences. The SC product has been submitted for a Chinese invention patent (application number: CN202011177498.0; public number: CN112341290A) [24].

Furthermore, the SC has been subjected to rigorous testing and demonstration in saline–alkali land in multiple locations over the years (https://www.163.com/dy/article/IJLS0P380518JU61.html, accessed on 16 November 2023) [25]. Thus, a more in-depth mechanism study was conducted in the present work, based on the tests of the yield effects in multiple locations over many years.

### 2.2. Experimental Site

The experiment was conducted at the National Salt-Tolerant Rice Technology Innovation Center, Dadan Village, District Yazhou, Hainan Province, China, from December 2021 to May 2022 (109°16′ E, 18°36′ N). The experimental site was flat with uniform fertility that remained under paddy cultivation but was kept fallow in the previous season. The experimental soil was sandy soil, and the physicochemical properties of the soil before and after were determined according to Wang et al. [26], and the Na^+^ was then determined using a flame photometer [9]. The details of the physicochemical properties of the soil are shown in Table S1.

### 2.3. Experimental Treatments and Crop Management

The experimental treatments were composed of two hybrid rice varieties, i.e., ‘Y Liangyou 957’ (YLY957) and Jing Liangyou 534 (JLY534), and two soil amendment treatments, i.e., the application of SC at control levels and 2250 kg hm^−2^, named ‘CK and SC’, respectively, and were arranged in a randomized complete block design. The hybrid rice varieties, i.e., Y Liangyou 957 (YLY957) and Jing Liangyou 534 (JLY534), were provided by the Hunan Hybrid Rice Research Center, Hunan, China. The cultivar type, parent source, and maturity period are shown in Supplementary Table S2. Ridges (50 cm) were built between the plots and wrapped with plastic film to prevent water and fertilizer runoff. The seeds of both varieties were sown on 16 December 2021 and transplanted manually on 16 January 2022 (seedling age: 31 days) with plant spacing of 15 cm × 30 cm. The crop was applied with a mixture of underground and sea water having an EC value of 11 dS/m. Each treatment was replicated 3 times, with 12 experimental units, each with an area of approximately 20 m^2^. Then, 210 kg hm^−2^ of N, 168.75 kg hm^−2^ of P_2_O_5_, and 210 kg hm^−2^ of K_2_O were applied to all treatments throughout the whole growth period, whereas the SC treatment was combined with an additional dose of 2250 kg hm^−2^ of soil conditioner and 375 kg hm^−2^ of 42% (18-6-18) compound fertilizer. Tiller fertilizer was applied after transplanting, with 46.2% urea 75 kg hm^−2^ and 60% KCl 69 kg hm^−2^. Spike fertilizer was applied with 46.2% urea 136.5 kg hm^−2^ and 60% KCl 37.5 kg hm^−2^. The specific fertilizers for the CK were as follows: basal fertilizer was applied with 42% (18-6-18) compound fertilizer 585 kg hm^−2^ and 12% calcium superphosphate 1001.25 kg hm^−2^. The tillering fertilizer was applied with 46.2% urea 91.5 kg hm^−2^ and 60% KCl 70.5 kg hm^−2^. The spike fertilizer was applied with 42% (18-6-18) compound fertilizer at 225 kg hm^−2^, 46.2% urea at 49.5 kg hm^−2^, and 60% potassium chloride at 37.5 kg hm^−2^. After transplanting, fresh water was applied for irrigation for 26 days for better stand establishment. Later on, irrigation with 0.6% saline water (sea water and fresh water mixed in a mixing pool to 0.6%) was started, maintaining a water layer of about 5 cm throughout the growth period. Pest, disease, and weed control was carried out in accordance with the provincial government’s guidelines for rice cultivation.

### 2.4. Determination of Leaf SPAD Value, Leaf Area Index, and Dry Matter Accumulation

The SPAD values were measured at the heading stage (HS) and 15 days after the HS using a portable chlorophyll meter (SPAD-502 PLUS, Konica Minolta, Tokyo, Japan). In brief, five uniform plants from each replicate were selected and taken at the midpoint of the sword leaf, 1/2 to 1/3 from the leaf tip, and averaged. Subsequently, eight representative plants were selected from each plot, and the green leaves and stem sheaths (including panicle) were separated. The aboveground leaf area was determined using the Li-COR3100 (LI-COR, Lincoln, NE, USA), and the stem sheath samples were dried in an oven at 75 °C to a constant weight. The leaf area index and dry matter accumulation per unit area of leaves and stem sheaths were measured. The dry matter translocation and the translocation rates of the leaves and stem sheaths were calculated as follows:

Dry matter translocation in the leaf/stem sheath (t hm^−2^) = dry matter accumulation in the leaf/stem sheath at HS − MS

Dry matter translocation rate (%) = dry matter translocation in leaf (stem sheath)/dry matter accumulation in leaf/(stem sheath) at HS × 100

### 2.5. Antioxidant Enzyme Activity and Malondialdehyde (MDA) Content

At the HS, fresh leaves were taken from the whole plant from each plot and stored at −80 °C for the determination of the antioxidant enzyme activity, i.e., superoxide dismutase (SOD), peroxidase (POD), and catalase (CAT), and the MDA content at 30 days at the HS. The antioxidant enzyme activity and MDA were estimated according to Li et al. [27].

### 2.6. Yield and Yield Components

Rice plants (12 hills from each plot) were sampled at the MS and the effective panicle number, grains per panicle, seed setting rate, and 1000-grain weight were determined. The grain yield of each plot was measured from a 3 m^2^ sampling area with standard grain moisture content of 13.5%.

### 2.7. Grain Quality

The grains were threshed manually and air-dried at room temperature to water content of 12%. Subsequently, a portion of the rice was stored at room temperature for a duration of 3 months, while another portion was stored at 4 °C for subsequent physio-biochemical analyses [28]. Rice grains (200 g) from the stored grains were taken and a rice huller (Thu35c, Satake, Hiroshima, Japan) and Jingmi rice grader (Zhejiang Top Cloud-Agri Technology Co., Ltd., Zhejiang, China) were used to obtain the milled rice, whereas a sieve (10-mesh) was used to remove broken rice grains. The head rice samples (50 g) were taken to determine the taste value using a rice taste meter (STA1A, Satake, Hiroshima, Japan). The gel consistency and amylose starch content were determined according to the agricultural industry standards of China, NY/T 83-2017 and NY/T 2639, respectively [29]. The protein content was determined according to Li et al. [9]. The total starch content was determined using a total starch detection kit (Megazyme, K-TSTA, Beijing, China).

### 2.8. Starch Isolation, Starch Size, and Starch Granule Morphology

Starch isolation was conducted according to Jin et al. [30], whereas the starch particle size distribution was analyzed using a laser particle sizer (Mastersizer 3000, Malvern Instruments Ltd., Worcestershire, UK). The starch granule morphology was then observed by high-resolution field emission SEM (Zeiss Merlin Compact, Zeiss, Oberkochen, Germany).

### 2.9. Pasting Property and Thermal Characteristics Analysis

The starch pasting properties and thermal properties were measured according to Yao et al. [31]. The starch pasting properties were analyzed using a Rapid Viscosity Analyzer (RVA) (RVA Super 4, Newport Scientific, Warriewood, NSW, Australia) and the thermal properties were measured using a differential scanning calorimeter (Q2000, TA Instruments, New Castle, DE, USA).

### 2.10. Data Analysis

A one-way analysis of variance (ANOVA) was conducted on the collected data using the SPSS 19.0 software (SPSS, Inc., Chicago, IL, USA). The differences among the treatment means of each experiment were separated using Tukey’s post hoc test at a significance level of 0.05. All graphs were created using Origin 9.0 (OriginLab Corp., Northampton, MA, USA).

## 3. Results

### 3.1. Rice Yield and Its Component Factors

The application of SC substantially affected the grain yield and spikelets per panicle, as well as increasing the yield to 27.6% and 23.3% in both rice varieties, i.e., YLY957 and JLY534, respectively. Moreover, the spikelets per panicle for YLY957 and JLY534 were increased by 29.6% and 28.4%, respectively, in the SC treatment, whereas no significant difference between the CK and SC treatments was noticed in terms of the effective panicle number and 1000-grain weight for both rice varieties. However, SC application increased the grain filling rate of JLY534 by 5.0%. The improvement in grain yield traits ultimately led to an increase in the overall yield of both hybrid rice varieties (Table 1).

### 3.2. Agronomic Traits

**Figure 1:**
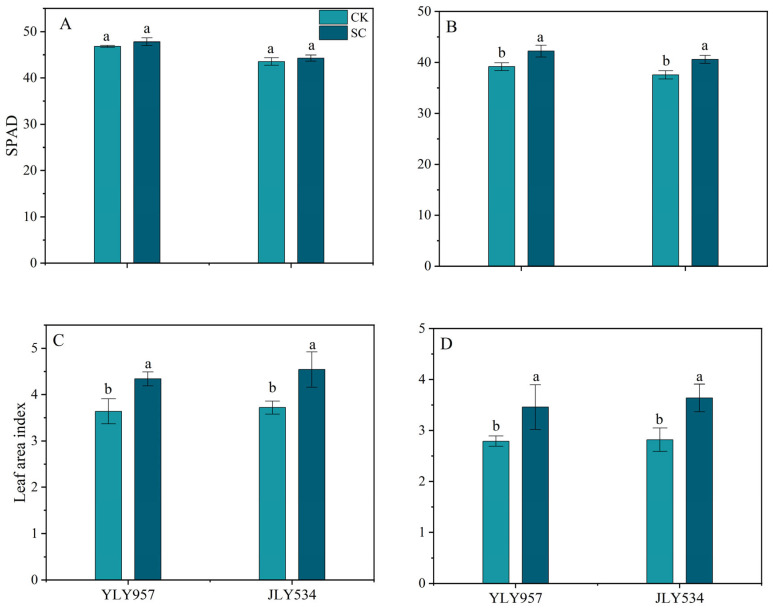
Effects of soil conditioner on SPAD and leaf area index during heading (**A**,**C**) and 15 days after heading stage (**B**,**D**) under saline conditions. Values ± SD (n = 3) of the same cultivar with different letters are significantly different (*p* < 0.05). YLY957, Y Liangyou 957; JLY534, Jing Liangyou 534; CK, control; SC, soil conditioner.

SC application variably affected the leaf area index and SPAD at the HS and 15 days after the HS (Figure 1). No significant difference between the CK and SC treatments was found regarding the SPAD; however, the SC significantly improved the leaf area index by 19.2% and 22.0% for YLY957 and JLY534, respectively, at the HS. In addition, the SC significantly increased the leaf area index and SPAD of both varieties at 15 days after the HS, whereas, for JLY534, the SC increased the leaf area index and SPAD by 29.1% and 8.1%, respectively, being comparatively higher than those of YLY957.

Similarly, SC application had different effects on the dry matter accumulation of different parts of both rice varieties at the HS and MS (Table 2). At the HS, the stem sheaths of YLY957 and JLY534 were increased by 6.3% and 7.0%, respectively, in the SC treatment compared to the CK. At maturity, the stem sheath weight was reduced by 5.3% and 10.3%, respectively, in the SC treatment as compared to the CK. Furthermore, at the HS, the leaf was enhanced by 12.5% and 26.7%, respectively, with SC application compared to the CK, while the SC also improved the total dry matter accumulation of both rice varieties substantially at the HS and MS.

**Figure 2:**
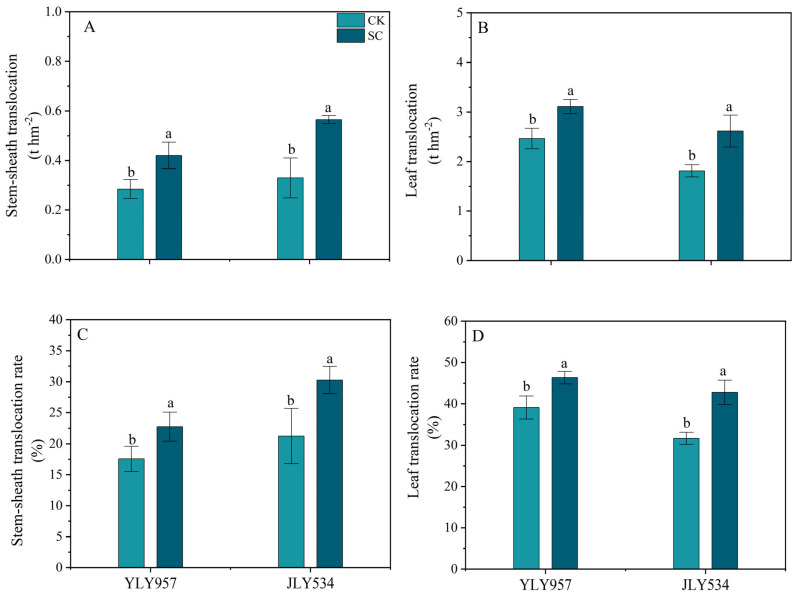
Effects of soil conditioner on stem sheath (**A**) and leaf translocation (**B**) and stem sheath (**C**) and leaf translocation rate (**D**) from heading to maturity under saline conditions. Values ± SD (n = 3) of the same cultivar with different letters are significantly different (*p* < 0.05). YLY957, Y Liangyou 957; JLY534, Jing Liangyou 534; CK, control; SC, soil conditioner.

In addition, SC application increased the amount and rate of biomass translocation in the leaves and stem sheaths at the HS and MS (Figure 2). Specifically, compared with the CK, the SC treatment significantly increased the stem sheath and leaf translocation amount of biomass by 26.3% and 44.4% and by 47.7% and 71.5% in YLY957 and JLY534, respectively. On the other hand, the biomass translocation rate in the stem sheaths and leaves of YLY957 was increased by 46.4% and 22.8% and by 18.5% and 29.4%, respectively, in the SC treatment as compared with the CK. Additionally, the increase in the biomass translocation rate in YLY957 was lower than that in JLY534.

### 3.3. Antioxidant Enzymes and Malondialdehyde (MDA) Content

**Figure 3:**
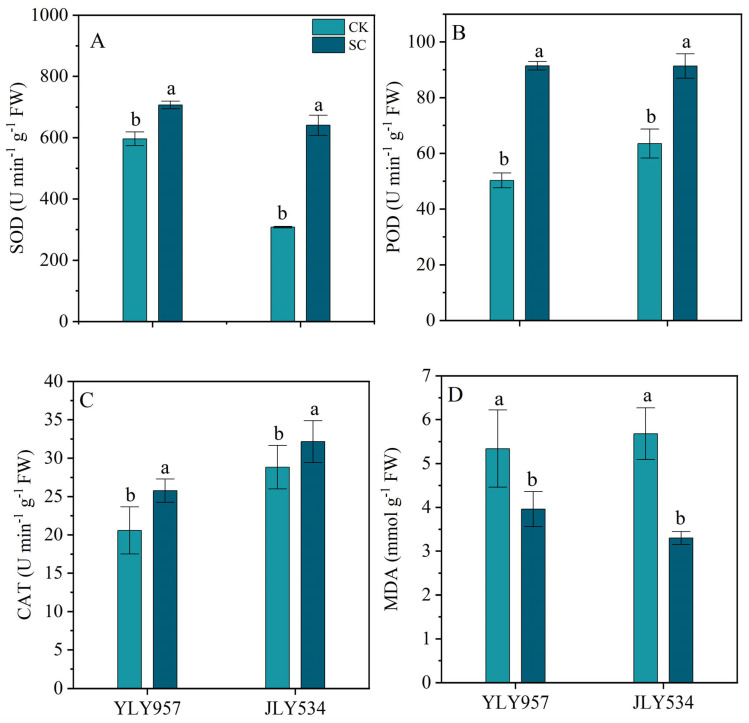
Effects of soil conditioner on SOD (**A**), POD (**B**), CAT (**C**) and MDA content (**D**) of flag leaves under saline conditions. Values ± SD (n = 3) of the same cultivar with different letters are significantly different (*p* < 0.05). YLY957, Y Liangyou 957; JLY534, Jing Liangyou 534; CK, control; SC, soil conditioner. SOD, superoxide dismutase; POD, peroxidase; CAT, catalase; MDA, malondialdehyde.

The application of SC substantially improved the activity of antioxidant enzymes in YLY957 and JLY534 at the HS (Figure 3). For example, the SOD activity in YLY957 and JLY534 treated with SC increased by 18.5% and 107.8%, respectively, while the POD activity increased by 81.8% and 43.8%, respectively, compared to the CK. Additionally, the CAT activity in YLY957 and JLY534 subjected to the SC increased by 25.2% and 11.5%, respectively, under saline conditions. Furthermore, the SC treatment significantly reduced the malondialdehyde content in YLY957 and JLY534 by 25.8% and 41.9%, respectively.

### 3.4. Milling Quality and Appearance Quality of Rice

The application of SC improved the rice quality, especially in terms of increasing the milling rice rate and head rice rate, and reduced the chalkiness degree (Table 3). The head rice rate was 1.4% and 2.5% higher in the SC treatment than the CK for YLY957 and JLY534, respectively. Moreover, SC application reduced the chalkiness degree by 1.6% to 0.9% but decreased the chalky grain rate from 5.0% to 3.7% in YLY957. The application of SC also increased the milling rice rate and head rice rate of JLY534 by 1.4% and 3.9%, respectively, while the chalkiness degree and chalk grain rate were significantly reduced.

### 3.5. Chemical Composition and Taste Value

SC application substantially improved the total starch and amylose content, paste viscosity, and taste value of both rice varieties and reduced the protein content significantly (Table 4). The total starch and amylose content increased by 4.6% and 36.9% (in YLY957) and 2.4% and 32.8% (in JLY534), respectively, with SC application, as compared with the CK. Compared with the CK, the protein content in YLY957 and JLY534 was decreased by 29.1% and 25.6%, respectively, whereas the paste viscosity and taste value were increased by 10.0–13.2% and 31.2–15.2%, respectively, for both varieties with SC application compared to the CK.

### 3.6. RVA and Gelatinization Characteristics

The SC had a significant impact on the RVA characteristics of both rice varieties (Table 5). The peak viscosity (PV), trough viscosity (TV), final viscosity (CPV), and breakdown viscosity (BD) were increased by 35.4%, 26.5%, 21.7%, and 59.1% (in YLY957) and 17.4%, 19.3%, 13.3%, and 13.1% (JLY534), whereas the setback viscosity (SB) and pasting temperature (PT) were decreased by 71.6% and 2.0% (in YLY957) and 8.8% and 0.5% (in JLY534) in the SC treatment as compared with the CK. Overall, the SC treatment substantially improved the quality characteristics of rice in both varieties, especially improving the viscosity and stability.

In addition, the SC treatment had a significant impact on the gelatinization characteristics of the rice, specifically in reducing the gelatinization temperature and enthalpy (Table 6). For YLY957, no significant difference was noticed in the onset temperature, peak temperature, and final temperature between the SC and CK treatments; however, the SC treatment significantly reduced the onset temperature, peak temperature, and final temperature, with an increase of 1.8%, 1.8%, and 2.8%, respectively, in JLY534. In addition, SC significantly reduced the enthalpy value of YLY957 and JLY534 by 8.3% and 11.1%, respectively, as compared with the CK.

### 3.7. Starch Granule Size of Rice

**Figure 4:**
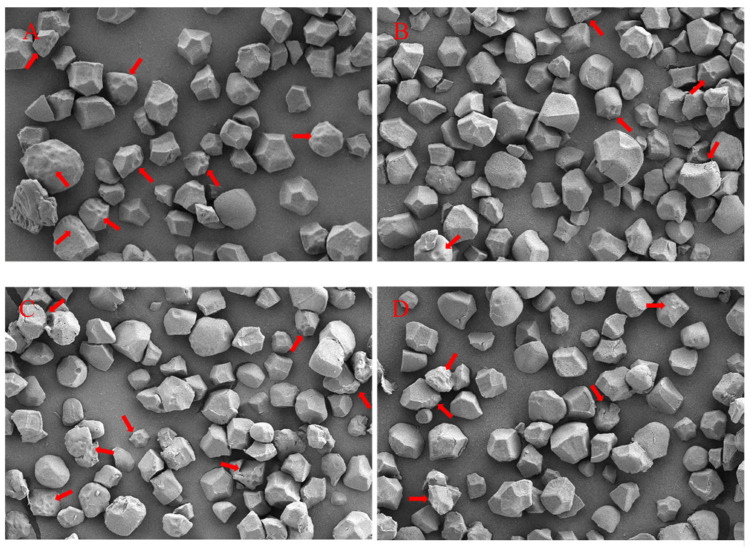
Scanning electron microscopy micrographs of purified starch. (**A**,**B**) are the starch of YLY957 under the control and soil conditioner, respectively. (**C**,**D**) are the starch of JLY534 under the control and soil conditioner, respectively. The magnification ratio of Figure 4 is 5000. The arrows indicate changes (indentations and cracks).

The application of SC also decreased the indentations on the surfaces of the starch granules and cracks on the edges of the granules, compared to the CK (Figure 4). The volume, surface area, and number of starch granules were variably affected by SC application (Table 7). For YLY957, the volume, surface area, and number of starch granules (with a diameter < 10 μm) were reduced by 2.6%, 17.8%, and 12.2%, respectively, with SC application. The reductions in the volume, surface area, and number of starch granules (with a diameter of <10 μm) in YLY957 were greater than those in JLY534. However, the opposite results were found for a diameter > 10 μm, where the SC treatment significantly increased the volume, surface area, and number of starch granules with a diameter > 10 μm for both rice varieties.

### 3.8. Amylopectin and Crystallinity of Rice

**Figure 5:**
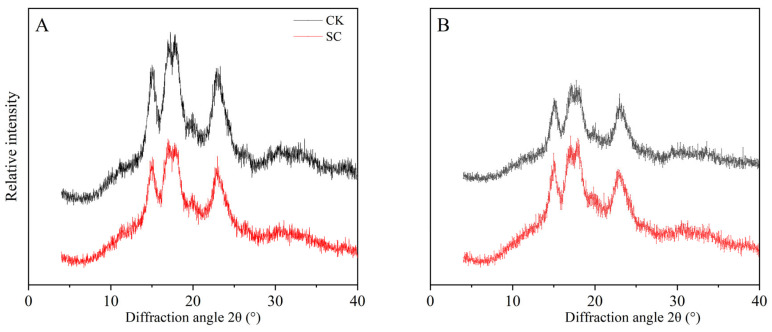
Effects of soil conditioner on the X-ray diffraction patterns of rice starch for cultivars Y Liangyou 957 (YLY957) (**A**) and Jing Liangyou 534 (JLY534) (**B**) under saline conditions (n = 3).

The SC treatment had a significant impact on the distribution of the amylopectin chain lengths and the crystallinity of both rice varieties (Table 8; Figure 5). Compared with CK, the SC treatment reduced the relative crystallinity by 2.8% in YLY957 and increased it by 11.9% in JLY534. In addition, all samples had an A-type crystallization pattern, whereas the SC treatment did not change the crystallization pattern. Moreover, the SC treatment decreased the DP 6–12 chain length (A) by 6.5% and 4.4% and increased the DP 13–24 (B1) by 9.8% and 12.0% in YLY957 and JLY534, respectively. Furthermore, the SC treatment significantly decreased the DP 25–36 (B2) and DP ≥ 37 (B3) of both rice varieties.

### 3.9. Soil Nutrients

In addition to the total nitrogen (N) content, the SC treatment showed higher content for all other indicators, especially the organic matter, total potassium (K), available phosphorus (P), and available K content, which were increased by 19.9%, 32.4%, 55.2%, and 13.4%, respectively, in the SC treatment as compared to CK. In addition, no significant difference in the sodium ions, total P, and available nitrogen (N) was noted for the SC and CK treatments (Table 9).

## 4. Discussion

Generally, soil is considered to be salinized if the content of water-soluble salts in the surface and/or subsurface soil layer is 0.1% and/or if the soil alkalinity exceeds 5% [15]. The excessive accumulation of salts interferes with crop nutrients and ultimately leads to plant death [3]. Soil microbial communities are not only diverse and numerous but also possess outstanding adaptability, which enables them to thrive and function in different ecological niches, where other forms of life struggle to adapt. Soil microbes also play a key role in the natural cycles of matter and energy and have a significant role in alleviating the salinity problems in saline–alkali areas [32]. Compared to other salt amelioration techniques, the microbial salt amelioration strategy is highly efficient and environmentally friendly, making it an important tool in promoting sustainable development in coastal saline–alkali lands, and it is now becoming an interesting topic of research on soil reclamation programs [33]. This study explored the impact of salt-tolerant microorganism-based soil conditioners on the growth, yield, quality, and physicochemical and structural properties of starch grains of hybrid rice under saline stress conditions.

In the present study, it was found that salt stress had a severely negative impact on the spikelets per panicle, which is possibly a significant factor for reduced yields in rice under saline conditions. Previously, Guo et al. [8] also reported that a decrease in the number of effective panicles and the number of grains per panicle in rice under salt stress is the basic reason for a rice yield reduction. Furthermore, Chen et al. [7] found that salt stress primarily affected the seed setting/filling rate and the number of grains per panicle, regardless of whether the rice was salt-tolerant or sensitive to salinity. Nevertheless, the present study showed that the SC’s application mainly enhanced the rice yield by improving the number of grains per panicle in both hybrid rice cultivars, whereas the higher yield with SC is mainly due to differences in agronomic and physiological traits. For example, the SC treatment maintained a better leaf area index and SPAD during the 15 days after the HS, delayed leaf senescence, and ensured better photosynthate transport and accumulation. In addition, the SC treatment promoted the transport of photosynthetic products from the rice leaves and sheaths to the grains, thereby inhibiting the degeneration of small panicles and ensuring a higher number of grains per panicle and filling rate [9,34]. Moreover, our results indicate that the SC treatment promoted the activity of antioxidant enzymes in the leaves during the HS stage, thereby reducing the MDA content under salt stress conditions. Overall, the SC treatment improved the morpho-agronomic traits and enhanced the salt tolerance of rice, which may have been related to its microbial composition [32]. Similarly, Ding et al. [14] found that the objective of microbial-based biofertilizers is the introduction and multiplication of the microbial communities in soil, which can rapidly interact with soil-beneficial microorganisms to form a dominant bacterial community in the soil, thereby alleviating salt stress. Wang et al. [35] found that the number of bacteria significantly increased after the application of microbial biofertilizers, possibly owing to the fact that the bacteria in the soil are initially the main components of the soil microbial flora. In addition, Zheng et al. [18] found that salt-tolerant leguminous plants, such as wild soybeans, promote beneficial microbial groups by secreting key metabolites to resist salt stress, thereby ensuring good agronomic and yield traits. Therefore, the application of microorganism-based soil conditioners can improve the salt tolerance of rice through maintaining soil microbial interactions with improved rice growth and yields.

Additionally, SC application enhanced the salt tolerance of rice by increasing the soil organic matter, total P, available P, and available K content, which is consistent with a previous study [36] that suggests that soil conditioners can potentially improve the soil structure and crop stress resistance. SC application increased the soil organic matter and P and K content, which was mainly related to the crop straw content in the SC. Previous studies have shown that straw application can improve the soil fertility and increase the soil NPK content [37]. Hassani et al. [38] analyzed 43,459 mineral soil samples collected from different land cover types since 1992 and found that the soil salinity was negatively correlated with the soil organic carbon variability. Therefore, ensuring the soil organic matter content is a beneficial measure to reduce soil salinity. Ding et al. [14] found that the application of biofertilizers significantly increased the available N, P, and K in the soil and significantly reduced the soil electrical conductivity compared to the application of inorganic fertilizers, which is consistent with our results. Saline–alkali soils contain high levels of Na^+^, leading to a low K^+^/Na^+^ ratio in the soil. Due to the similarity in the ionic radius and hydration energy, Na^+^ can compete with K^+^ for binding sites [39]. The low K^+^/Na^+^ ratio in the soil solution inhibits the absorption and utilization of K^+^ by crops, leading to K^+^ deficiency in crops under salt stress [40]. Furthermore, Sun et al. [41] found that increasing the K^+^ content in the soil promotes the absorption and utilization of K^+^, meeting the crop’s potassium needs while alleviating salt stress damage. Therefore, the application of SC not only improves the salt tolerance of rice but also promotes the soil nutrient content and soil organic matter, providing a basis for soil improvement.

Previous studies have also shown that salt stress reduces the milling and appearance quality of rice [34]. The appearance quality of rice includes the grain size and shape, chalkiness, and transparency, with chalkiness being the most important as it reduces the visual appeal of rice grains and affects consumer preferences [42]. In addition, the proportions of brown rice, milled rice, and head rice are economically important quality traits of rice [43]. Salt stress inhibits the nutrient supply, especially during the grain filling period, and limits the translocation of photosynthetic products to the grains, which results in the loose arrangement of starch granules in the endosperm and the formation of cavities, leading to grain chalkiness [44,45]. Our study also showed that the application of SC reduced grain chalkiness and improved the milled and head rice rates. Moreover, Lanning et al. [46] reported that the starch granules in the chalky areas of rice grains exhibit a blocky or granular structure with porous and loose arrangement characteristics, leading to a reduction in the toughness and milling quality of the grains, which corroborates our results. We further found that the chalkiness was significantly and negatively correlated with the milled and head rice rates, while the application of SC ensured a sufficient nutrient supply during the heading and filling periods. Therefore, the application of SC can ensure the coordinated improvement of the processing and appearance quality of hybrid rice, thereby ensuring the economic benefits of rice under salt stress conditions.

The cooking and eating quality attributes of rice are profoundly influenced by its starch composition, which includes amylose and amylopectin, as well as the presence of proteins [43,45]. Jin et al. [30] reported that the exposure of rice to saline conditions can elevate the protein levels in grains while simultaneously diminishing the starch content and thus the palatability as well. Similarly, other studies have also shown that salt stress reduces the eating quality of rice by increasing the grain protein content [31,43]. However, Cui et al. [47] found that the protein content decreased under low salt stress conditions and increased under high salt stress conditions. We speculate that the increase or decrease in protein under salt stress is closely related to the degree of salt stress. In the present study, the application of the SC treatment notably augmented the levels of total starch and amylose, while concurrently lowering the protein content. Higher protein and amylose content increase the stability of the starch’s crystal structure and heat resistance, which limits the expansion and leaching of starch during rice cooking and results in a harder, less sticky texture in rice [48]. Furthermore, an elevated protein concentration hinders the absorption of water by starch granules, which adversely affects the rice flavor [49]. Rice varieties that are resilient to salt stress are characterized by lower amylose and protein content, a reduced ratio of amylose to amylopectin under salt stress, and enhanced amylopectin and overall starch content, which collectively contribute to an enhanced taste profile [44]. On the other hand, high gel consistency is instrumental in enhancing the viscosity and firmness of rice, which in turn improves the taste value. The quality of rice when cooked and consumed is intricately tied to the RVA profile of starch. Typically, a superior taste is indicated by higher peak viscosity and breakdown values, contrasted with a lower setback value. Jin et al. [30] found a significant reduction in peak viscosity and breakdown values under salinity, which reduced the rice’s cooking quality. Conversely, in our study, the application of the SC treatment led to a marked increase in both the peak viscosity and breakdown values, thus substantially improving the culinary taste quality.

Jin et al. [30] underscore the pivotal role of the intricate architecture of amylopectin and its chain length distribution in shaping the culinary and gustatory traits of rice. The precise arrangement and span of the amylopectin chains are found to be closely linked to the physicochemical attributes of starch and its crystalline framework, which are instrumental in dictating the gelatinization behavior and thermal properties of starch during the cooking process [50]. According to Yao et al. [31], the imposition of saline conditions can reconfigure the starch composition in rice, notably altering the distribution patterns of the amylose and amylopectin chain lengths. Notably, the salt-tolerant rice varieties exhibited the increased presence of intermediate and extended amylopectin chains under saline conditions, which generally foster the formation of a more robust double helix conformation that influences the crystalline integrity and gelatinization profile of starch. Jin et al. [30] have also reported that saline conditions can induce alterations in the molecular and extensive structure of starch. Furthermore, it has been observed that salt stress is associated with a reduction in the proportion of short amylopectin chains (designated as A chains and B1 chains), while concurrently promoting an escalation in the prevalence of extended amylopectin chains (B2 chains and B3 chains). Our study revealed that the SC intervention was associated with an increase in B1 chains and the diminished presence of extended amylopectin chains (B2 chains and B3 chains). These shifts are supposed to exert effects on the crystalline configuration and the overall stability of the starch, which may subsequently alter the sensory attributes and mouthfeel of the rice.

The temperature at which starch undergoes gelatinization is indicative of the ease with which rice can be cooked, where fully processed rice grains tend to necessitate elevated temperatures and extended periods for cooking when developed under saline conditions [51]. Yao et al. [31] suggested that an increase in the gelatinization temperature is likely to be intricately linked to a reduction in the quantity of amylopectin and the proportion of its short chains, juxtaposed with an augmentation in the number of medium and long chains. An abundance of long chains is capable of creating extensive double helix configurations, which in turn augment the intermolecular interactions and reinforce the crystalline matrix of starch; this restricts the ability of the starch to swell during gelatinization, necessitating greater thermal energy for its disintegration and consequently yielding higher paste viscosities [47]. Moreover, the heat enthalpy value, denoted as (AHgel), exhibits a positive association with both the gelatinization temperature and the degree of crystallinity [31]. Our study revealed that the application of the SC led to a reduction in the gelatinization temperature with a decrease in crystallinity, primarily attributed to a reduction in the prevalence of extended chains (B2 + B3) within amylopectin. It has been suggested that salt stress catalyzes the solidification of a robust crystalline framework within the rice starch and necessitates an intensified thermal input for gelatinization [43]. Jin et al. [30] have reported shifts in the starch granule size distribution, particularly noting an increase in the prevalence of finer starch particles (diameter < 10 μm), whereas salt stress is also implicated in diminishing the relative crystallinity of starch. Our findings further indicate that SC application reduced the proportion of fine starch particles (diameter < 10 μm), thereby attenuating the relative crystallinity, which suggests that the incorporation of SC modulates the integrity of the crystalline structure of starch through the modulation of the chain lengths and starch distribution, which in turn ameliorates the overall quality of hybrid rice.

## 5. Conclusions

In summary, the application of SC improved the yield and salt tolerance of hybrid rice by regulating the soil organic matter and nutrient content and the antioxidant defense system and strengthening the source–sink relationship. The application of SC also improved the appearance and taste quality of the rice, optimized the physicochemical properties of the rice starch, and increased the peak viscosity and breakdown value, while reducing the setback value and gelatinization temperature, demonstrating better gelatinization performance. This work not only provides effective strategies for the agricultural sustainability of saline soils but also highlights potential application prospects for other crops that are prone to soil salinization. Future research can further explore the combined use of different soil conditioners and their effects under various levels of salinity, with the aim to provide more comprehensive and in-depth solutions for saline agriculture.

## Figures and Tables

**Table 1:** Effects of soil conditioner on rice yield and its component factors under saline conditions.

Cultivar	Treatment	Productive	Spikelets	Grain	1000-Grain	Grain
		Panicle	per	Filling	Weight	Yield
		(m^−2^)	Panicle	(%)	(g)	(t hm^−2^)
YLY957	CK	191.3 ± 7.7 a	111.6 ± 9.1 b	80.5 ± 3.5 a	16.3 ± 0.1 a	2.9 ± 0.1 b
	SC	191.2 ± 14.4 a	144.7 ± 4.2 a	81.8 ± 2.4 a	16.3 ± 0.2 a	3.7 ± 0.1 a
JLY534	CK	211.6 ± 5.8 a	106.0 ± 9.3 b	79.8 ± 0.3 b	15.8 ± 0.1 a	3.0 ± 0.1 b
	SC	207.3 ± 10.1 a	136.1 ± 3.2 a	83.8 ± 1.4 a	16.1 ± 0.1 a	3.7 ± 0.2 a

Values ± SD (n = 3) in the same column of the same cultivar with different letters are significantly different (*p* < 0.05). YLY957, Y Liangyou 957; JLY534, Jing Liangyou 534; CK, control; SC, soil conditioner.

**Table 2:** Effect of soil conditioner on leaf, stem sheath, and total biomass at heading and maturity stages under saline conditions.

Cultivar	Treatment	Stem Sheath		Leaf		Panicle	Total Biomass	
		Weight (t hm^−2^)		Weight (t hm^−2^)		(t hm^−2^)	(t hm^−2^)	
		Heading	Maturity	Heading	Maturity	Maturity	Heading	Maturity
YLY957	CK	6.3 a	3.8 a	1.6 b	1.3 a	3.0 b	7.9 b	8.1 b
	SC	6.7 a	3.6 a	1.8 a	1.4 a	3.8 a	8.5 a	8.8 a
JLY534	CK	5.7 a	3.9 a	1.5 b	1.2 a	2.8 b	7.3 a	7.9 b
	SC	6.1 a	3.5 b	1.9 a	1.3 a	3.7 a	8.0 b	8.5 a

Values ± SD (n = 3) in the same column of the same cultivar with different letters are significantly different (*p* < 0.05). YLY957, Y Liangyou 957; JLY534, Jing Liangyou 534; CK, control; SC, soil conditioner.

**Table 3:** Effect of soil conditioner on processing quality and appearance quality of rice under saline conditions.

Cultivar	Treatment	Brown Rice	Milled Rice	Head Rice	Chalkiness	Chalky Grain
		Rate (%)	Rate (%)	Rate (%)	Rate (%)	Rate (%)
YLY957	CK	80.1 ± 0.6 a	72.8 ± 0.2 b	68.6 ± 0.8 b	1.6 ± 0.2 a	5.0 ± 1.0 a
	SC	81.3 ± 0.2 a	73.8 ± 0.3 a	70.3 ± 1.1 a	0.9 ± 0.1 b	3.7 ± 0.6 a
JLY534	CK	79.9 ± 0.5 a	72.7 ± 0.3 b	68.9 ± 0.3 b	1.0 ± 0.2 a	4.3 ± 0.6 a
	SC	80.4 ± 0.3 a	73.7 ± 0.3 a	71.6 ± 0.5 a	0.7 ± 0.1 b	2.7 ± 0.6 b

Values ± SD (n = 3) in the same column of the same cultivar with different letters are significantly different (*p* < 0.05). YLY957, Y Liangyou 957; JLY534, Jing Liangyou 534; CK, control; SC, soil conditioner.

**Table 4:** Effects of soil conditioner on nutritional quality and eating quality of rice under saline conditions.

Cultivar	Treatment	Total Starch	Amylose	Protein	Gel	Taste
		Content	Content	Content	Consistency	Value
		(%)	(%)	(%)	(mm)	
YLY957	CK	70.0 ± 1.4 b	10.3 ± 0.4 b	14.1 ± 0.5 a	67.0 ± 1.7 b	58.5 ± 2.7 b
	SC	73.2 ± 0.4 a	14.1 ± 0.2 a	10.0 ± 0.3 b	73.7 ± 0.6 a	62.6 ± 1.9 a
JLY534	CK	69.9 ± 1.1 b	11.9 ± 0.4 b	13.3 ± 0.6 a	57.7 ± 1.2 b	56.7 ± 2.9 b
	SC	71.6 ± 1.2 a	15.8 ± 0.5 a	9.9 ± 0.2 b	65.3 ± 0.6 a	65.3 ± 3.6 a

Values ± SD (n = 3) in the same column of the same cultivar with different letters are significantly different (*p* < 0.05). YLY957, Y Liangyou 957; JLY534, Jing Liangyou 534; CK, control; SC, soil conditioner.

**Table 5:** Effects of soil conditioner on starch pasting properties under saline conditions.

Cultivar	Treatment	PV (cP)	TV (cP)	CPV (cP)	BD (cP)	SB (cP)	PT (°C)
YLY957	CK	2030.0 b	1469.5 b	2326.0 b	560.5 b	296.0 a	92.3 a
	SC	2748.7 a	1856.7 a	2831.3 a	892.0 a	82.7 b	90.4 b
JLY534	CK	2111.5 b	1458.5 b	2498.5 b	653.0 b	387.0 a	93.0 a
	SC	2478.0 a	1739.3 a	2831.0 a	738.7 a	353.0 b	92.5 b

Values ± SD (n = 3) in the same column of the same cultivar with different letters are significantly different (*p* < 0.05). YLY957, Y Liangyou 957; JLY534, Jing Liangyou 534; CK, control; SC, soil conditioner; BD, breakdown viscosity; CPV, final viscosity; PT, pasting temperature; PV, peak viscosity; SB, setback viscosity; TV, trough viscosity.

**Table 6:** Effects of soil conditioner on starch gelatinization properties under saline conditions.

Variety	Treatment	Onset	Peak	Conclusion	Enthalpy
		Temperature	Temperature	Temperature	(J/g)
		(°C)	(°C)	(°C)	
YLY957	CK	76.3 ± 0.1 a	83.0 ± 0.6 a	87.4 ± 0.2 a	6.0 ± 0.2 a
	SC	76.4 ± 0.8 a	83.5 ± 0.3 a	87.6 ± 0.6 a	5.4 ± 0.1 b
JLY534	CK	69.2 ± 0.4 b	74.7 ± 0.4 b	78.9 ± 0.3 b	5.2 ± 0.2 a
	SC	70.5 ± 0.4 a	76.1 ± 0.4 a	81.2 ± 0.6 a	4.8 ± 0.1 b

Values ± SD (n = 3) in the same column of the same cultivar with different letters are significantly different (*p* < 0.05). YLY957, Y Liangyou 957; JLY534, Jing Liangyou 534; CK, control; SC, soil conditioner.

**Table 7:** Effects of soil conditioner on starch granule size distribution under saline conditions.

Cultivar	Treatment	Volume		Surface Area		Number	
		Percentage (%)		Percentage (%)		Percentage (%)	
		d < 10 μm	d > 10 μm	d < 10 μm	d > 10 μm	d < 10 μm	d > 10 μm
YLY957	CK	97.4 ± 0.1 a	2.6 ± 0.1 b	56.6 ± 1.2 a	43.4 ± 1.2 b	78.7 ± 0.7 a	21.3 ± 0.7 b
	SC	94.9 ± 0.3 b	5.1 ± 0.3 a	46.5 ± 1.8 b	53.5 ± 1.8 a	69.1 ± 1.4 b	30.9 ± 1.4 a
JLY534	CK	96.2 ± 0.3 a	3.8 ± 0.3 b	54.4 ± 1.9 a	45.6 ± 1.9 b	75.2 ± 1.3 a	24.8 ± 1.3 b
	SC	95.6 ± 0.2 b	4.4 ± 0.2 a	47.4 ± 1.7 b	52.7 ± 1.7 a	70.4 ± 1.3 b	29.6 ± 1.3 a

Values ± SD (n = 3) in the same column of the same cultivar with different letters are significantly different (*p* < 0.05). YLY957, Y Liangyou 957; JLY534, Jing Liangyou 534; CK, control; SC, soil conditioner.

**Table 8:** Effects of soil conditioner on starch amylopectin chain length distribution and degree of crystallinity under saline conditions.

Cultivar	Treatment	Relative	Crystal	DP 6–12	DP 13–24	DP 25–36	DP ≥ 37
		Crystallinity (%)	Pattern	(%)	(%)	(%)	(%)
YLY957	CK	21.5 ± 0.4 a	A	27.9 ± 0.6 a	47.1 ± 0.2 b	12.2 ± 0.2 a	12.8 ± 0.3 a
	SC	20.9 ± 1.9 a	A	26.1 ± 0.1 b	53.1 ± 0.2 a	11.0 ± 0.1 b	9.8 ± 0.1 b
JLY534	CK	22.5 ± 0.1 a	A	30.6 ± 0.1 a	44.0 ± 0.1 b	12.6 ± 0.1 a	12.7 ± 0.1 a
	SC	20.1 ± 0.9 b	A	29.3 ± 0.3 b	50.0 ± 0.1 a	11.2 ± 0.1 b	9.4 ± 0.3 b

Values ± SD (n = 3) in the same column of the same cultivar with different letters are significantly different (*p* < 0.05). YLY957, Y Liangyou 957; JLY534, Jing Liangyou 534; CK, control; SC, soil conditioner. DP 6–12, DP 13–24, DP25–36, and DP ≥ 37 represent A chains, B1 chains, B2 chains, and B3 chains, respectively.

**Table 9:** Effects of soil conditioner on soil nutrients after rice harvest under saline conditions.

Indicator		Treatment	
		CK	SC
Na^+^ content (g kg^−1^)		6.0 a	6.2 a
Organic matter (g kg^−1^)		17.1 b	20.5 a
Total nitrogen content (g kg^−1^)		1.1 a	1.1 a
Total phosphorus content (g kg^−1^)		0.74 a	0.75 a
Total potassium content (g kg^−1^)		20.7 b	27.4 a
Available nitrogen content (mg kg^−1^)		106.8 a	114.6 a
Available phosphorus content (mg kg^−1^)		52.2 b	81.0 a
Available potassium content (mg kg^−1^)		279.5 b	317.0 a

Values ± SD (n = 6) in the same column of the same cultivar with different letters are significantly different (*p* < 0.05). CK, control; SC, soil conditioner application.

## Data Availability

Dataset available on request from the authors.

## Supplementary Materials

The following supporting information can be downloaded at: https://www.mdpi.com/article/10.3390/plants13162325/s1, Table S1 Physical and chemical properties of the initial soil; Table S2: Information about the cultivars was used in the experiment.
