# Supplementary material for: Halotolerant Microorganism-Based Soil Conditioner Application Improved the Soil Properties, Yield, Quality and Starch Characteristics of Hybrid Rice under Higher Saline Conditions
Source: Plants (Basel). 2024 Aug 21;13(16):2325. doi: 10.3390/plants13162325 (PMC11359022; doi:10.3390/plants13162325)
Supplement: Supplementary file 1 [file plants-13-02325-s001.zip › plants-3124173-supplementary.pdf]

Table S1 Physical and chemical properties of the initial soil

Indicators	value
pH	6.59
Na^+ content (g kg^{-1})	4.79
Organic matter (g kg^{-1})	18.6
Total N content (g kg^{-1})	0.96
Total P content (g kg^{-1})	0.58
Total K content (g kg^{-1})	25.0
Available N content (mg kg^{-1})	96.9
Available P content (mg kg^{-1})	42.6
Available K content (mg kg^{-1})	241.5

Table S2: Information about the cultivars was used in the experiment

Cultivars	Cultivar type	Parent source	Maturity period	Source institution
YLY957	Indica-type two-line hybrid rice	Y58S(♀)×Chuanghui957(♂)	132	Hunan Yuanchuang Super Rice Technology Co., Ltd.
JLY534	Indica-type two-line hybrid rice	Jing4155S(♀)×R534(♂)	130	Yuan Longping Agricultural High-Tech Co., Ltd
